# Blocking the CD47-SIRPα interaction reverses the disease phenotype in a polycythemia vera mouse model

**DOI:** 10.1038/s41375-023-01903-2

**Published:** 2023-04-24

**Authors:** Veronika Lysenko, Patrick M. Schürch, Selma Tuzlak, Nicole Wildner-Verhey van Wijk, Larisa V. Kovtonyuk, Burkhard Becher, Markus G. Manz, Stefanie Kreutmair, Alexandre P. A. Theocharides

**Affiliations:** 1 Department of Medical Oncology and Hematology, University of Zurich and University Hospital Zurich, Comprehensive Cancer Center Zurich, Zurich, Switzerland; 2 Institute of Experimental Immunology, University of Zurich, Zurich, Switzerland

**Keywords:** Myeloproliferative disease, Immunosurveillance, Erythropoiesis, Innate immunity

## Abstract

Polycythemia vera (PV) is a hematopoietic stem cell neoplasm driven by somatic mutations in *JAK2*, leading to increased red blood cell (RBC) production uncoupled from mechanisms that regulate physiological erythropoiesis. At steady-state, bone marrow macrophages promote erythroid maturation, whereas splenic macrophages phagocytose aged or damaged RBCs. The binding of the anti-phagocytic (“don’t eat me”) CD47 ligand expressed on RBCs to the SIRPα receptor on macrophages inhibits phagocytic activity protecting RBCs from phagocytosis. In this study, we explore the role of the CD47-SIRPα interaction on the PV RBC life cycle. Our results show that blocking CD47-SIRPα in a PV mouse model due to either anti-CD47 treatment or loss of the inhibitory SIRPα-signal corrects the polycythemia phenotype. Anti-CD47 treatment marginally impacted PV RBC production while not influencing erythroid maturation. However, upon anti-CD47 treatment, high-parametric single-cell cytometry identified an increase of MerTK+ splenic monocyte-derived effector cells, which differentiate from Ly6C^hi^ monocytes during inflammatory conditions, acquire an inflammatory phagocytic state. Furthermore, in vitro, functional assays showed that splenic *JAK2* mutant macrophages were more “pro-phagocytic,” suggesting that PV RBCs exploit the CD47-SIRPα interaction to escape innate immune attacks by clonal *JAK2* mutant macrophages.

## Introduction

Polycythemia vera (PV) is a hematopoietic stem cell (HSC) neoplasm driven by somatic *JAK2* mutations, characterized by the overproduction of red blood cells (RBCs) uncoupled from mechanisms that regulate erythropoiesis [1]. Although significant progress has been made in defining the cell-intrinsic and functional properties of PV HSCs, less is known about cell-extrinsic factors that govern PV erythropoiesis.

Numerous studies have shown that bone marrow (BM) macrophages promote erythroid maturation within erythroblastic islands, whereas splenic Ly6C^hi^ monocytes and macrophages contribute to phagocytose aged or damaged RBCs [2–4]. Ly6C^hi^ monocytes can either differentiate into tissue-resident macrophages or, during inflammatory conditions, into monocyte-derived effector cells (Mdcs), including monocyte-derived macrophages and monocyte-derived dendritic cells, which acquire an inflammatory phagocytic phenotype [5–8]. The binding of the anti-phagocytic (“don’t eat me”) CD47 ligand expressed on RBCs to the SIRPα receptor on macrophages inhibits phagocytic activity, thereby protecting RBCs from premature engulfment. Moreover, mice devoid of intracellular SIRPα-mediated signaling due to a truncation of the intracellular SIRPα domain (hereafter called “*Sirpα* mutant mice”) present with mild anemia, splenomegaly due to red pulp expansion and reduced RBC half-life [9]. The physiologic downregulation of CD47 on senescent RBCs contributes to their clearance in the splenic red pulp [10–12]. In addition, the expression of the pro-phagocytic (“eat me”) signal, calreticulin (CALR), further permits the uptake of RBCs by splenic macrophages in the absence of CD47 [13].

Given the vast contribution of macrophages to the maturation and degradation of RBCs, clonal pathological disorders that sustain an inflammatory setting, such as polycythemia vera, can further impact their behavior and function. Studies have shown that the depletion of macrophages in a PV mouse model leads to the normalization of the erythroid compartment suggesting a role for macrophages in the pathogenesis of PV [14, 15]. Moreover, *JAK2*-mutated CD11b + splenic cells express and produce increased amounts of pro-inflammatory cytokines and chemokines [16], essential for the recruitment and activation of phagocytic cells. Together, this data suggests that PV mice’s RBC life cycle remains under the control of macrophages.

In this study, we explore the role of the CD47-SIRPα interaction on the life cycle of PV RBCs. Our results show that CD47-SIRPα-signaling in a PV mouse model due to either anti-CD47 treatment or loss of the inhibitory SIRPα-signal corrects the polycythemia phenotype. The expansion of splenic Mdcs and functionally more “pro-phagocytic” PV macrophages upon CD47 blockade in PV mice suggests that PV RBCs exploit the CD47-SIRPα interaction to escape innate immune attacks by *JAK2* mutant macrophages.

## Materials and methods

### Animal experiments

In order to generate mice with a PV phenotype, mice expressing the floxed human *JAK2-*V617F transgene (*FF1*) under the control of a tamoxifen-inducible promotor were crossed with SclCre (*SclCre;FF1*) mice as described previously [17, 18]. Cre-recombinase expression was induced by injecting tamoxifen (2 mg dissolved in 200ul corn oil; controls received only corn oil) intraperitoneally for 5 consecutive days. PV mice expressing a truncated form of SIRPα were obtained by crossing the *SclCre;FF1* mice with SIRPα-mutant mice (hereafter called “triple-transgenic mice”) [19]. The experiment with triple-transgenic mice was performed twice, and the number of mice used is indicated in the figure legend. For competitive repopulation assays, BM-derived cells from transgenic mice expressing green fluorescent protein (GFP; *UBC-GFP*) were combined at a 1:1 ratio with either BM cells of induced *SclCre;FF1* (*JAK2* mutant) or uninduced *SclCre;FF1* (WT) mice and transplanted into lethally irradiated 8- to 12-week-old C57BL/6 CD45.1 mice. The cantonal veterinary office (Zurich, Switzerland) approved all conducted animal experiments. PV mice with a hemoglobin level below 150 g/L were excluded from the experiment to ensure a homogeneous group with a PV phenotype. For triple-transgenic mice experiments, mice were randomly selected and assigned to groups based on genotype availability. For competitive repopulation experiments, mice were assigned while maintaining similar ratios of males and females per experiment. No blinding was performed.

### Anti-CD47 treatment of PV mice

Eight weeks post-transplantation, WT or *JAK2* mutant mice were treated intraperitoneally 3 times a week for 2 or 4 weeks with an anti-mouse CD47 mAb (clone MIAP410; BioXCell) or a mouse IgG1 isotype control (clone MOPC-21; BioXCell) at a dose of 200 μg/mouse. Mice were sacrificed 1 day after the last antibody injection. The 2-week treatment experiment was performed twice, while the 4-week treatment experiment was performed three times. The 2-week treatment experiment for analysis of spleens using high-dimensional single-cell flow cytometry and biotinylation assay to determine RBC half-life was performed once. The number of mice used is indicated in the figure legends.

### Blood analysis with automated cell counter ADVIA 2120

Peripheral blood (PB) obtained by sublingual vein bleeding was drawn into EDTA-coated tubes (Sarstedt). Complete blood counts were measured on an ADVIA 2120 as described [20]. PB of mice treated with either IgG1 or MIAP410 was isolated before therapy started, 2 weeks after treatment, and at terminal analysis. For blood counts of treated mice, we also included mice that were not terminally analyzed. PB of triple-transgenic mice was isolated 2.5, 5, and 10 weeks after tamoxifen induction.

### Flow cytometry

To assess the erythroid, the immune, the hematopoietic stem and progenitor cell compartment in the BM/spleen and the CD47 expression on RBCs, cells were stained with the following antibodies: anti-mouse CD45.2 (clone 104, Biolegend), anti-mouse Gr-1 (clone RB6-8C5, eBioscience), anti-mouse F4/80 (clone BM8, eBioscience), anti-mouse CD11b (clone M1/70, Biolegend), anti-mouse B220 (clone RA3-6B2, Biolegend), anti-mouse MHCII (clone M5/114.15.2, Biolegend), anti-mouse CD11c (clone N418, eBioscience), anti-mouse CD44 (clone IM7, BD Pharmingen), anti-mouse CD47 (clone miap301, eBioscience), anti-mouse CD150 (clone TC15-12F12.2, Biolegend), anti-mouse CD48 (clone HM48-1, Biolegend), anti-mouse CD117 (clone 2B8, Biolegend), anti-mouse Ter119 (clone TER-119, eBioscience), anti-mouse Ly-6A/E (clone D7, Biolegend), anti-mouse CD34 (clone RAM34, eBioscience), anti-mouse 41a (clone eBioMWReg30 (MWReg30), eBioscience), anti-mouse CD34 (clone A2F10, eBioscience), anti-mouse Gr-1 (clone RB6-8C5, eBioscience), anti-mouse B220 (clone RA3-6B2, eBioscience), anti-mouse CD3e (clone 145-2C11, eBioscience), anti-mouse CD4 (clone GK1.5, eBioscience), anti-mouse CD8a (clone 53-6.7, eBioscience), anti-mouse CD11b (clone M1/70, eBioscience), anti-mouse IL-7Ra (clone A7R34, eBioscience), Hoechst 33342 (Invitrogen), and Zombie Aqua™ Fixable Viability Kit (Biolegend). Stained cells were analyzed using the BD LSRFortessa™ cell analyzer.

### High-dimensional single-cell flow cytometry

Spleens of indicated mice were cut into small pieces and then incubated in 0.4 mg/mL Collagenase IV (Sigma) and 0.04 mg/mL DNAse I (Sigma) in HBSS (Gibco) and 10% FBS (Gibco) for 0.5 h at 37 °C. After digestion, cell suspension was homogenized with an 18 G needle and syringe, filtered through a 100 μm cell strainer and washed. Erythrocytes were lysed using 1 mL RBC lysis buffer (155 mM NH_4_Cl, 12 mM NaHCO_3_, 0.1 mM EDTA) for 5 min on ice. Samples were washed in PBS and then resuspended in Live Dead Fixable Blue mixture (Thermo Scientific, 1:400) and anti-CD16/32 antibody (Table 1). After a washing step, anti-mouse flow cytometric antibodies were used for surface staining (Table 1). Cells were incubated for 20 min at 4 °C. For secondary staining step, cells were incubated 20 min at 4 °C. After another washing step, samples were acquired on a Cytek Aurora (Cytek Biosciences). Quality control of the Cytek Aurora was performed daily as instructed by the manufacturer. For downstream analysis, dead cells and doublets were excluded using FlowJo v10 (TreeStar). Exported FCS files were then transformed with an inverse hyperbolic sine (arcsinh) function using the R environment (range 1000–10’000). To balance the influence of markers with different dynamic ranges, we performed channel-based percentile normalization using the 99.9th percentile of each marker across the whole dataset [21]. Two-dimensional UMAP (Uniform Manifold Approximation and Projection) projections were calculated using the umap package [22]. All FlowSOM-based clustering was performed on the whole dataset, and the results were overlaid on the dimensionality reduction maps [23]. All plots were drawn using the ggplot2 package.

**Table 1:** List of antibodies.

anti-mouse antibody	Supplier	Clone
B220	BD	RA3-6B2
CD11b	BD	M1/70
CD172a = Sirpa	BD	P84
CD44	BD	IM7
CD47	BD	miap301
CD88	BD	20/70
Ly6G	BD	1A8
MHCII	BD	M5/114.15.2
NK1.1	BD	PK136
SiglecF	BD	E50-2440
CD121a = IL-1R	BioLegend	JAMA-147
CD16/32	BioLegend	93
CD19	BioLegend	6D5
CD206	BioLegend	C068C2
CD38	BioLegend	90
CD45.2	BioLegend	104
CD64	BioLegend	X54-5/7.1
CD86 = B7.2	BioLegend	GL-1
CD90.2	BioLegend	53-2.1
CX3CR1	BioLegend	SA011F11
F4/80	BioLegend	BM8
LY-6C	BioLegend	HK1.4
PD-1 (CD279)	BioLegend	29F.1A12
Tim-4	BioLegend	RMT4-54
VCAM-1	BioLegend	429
CD11c	Invitrogen	N418
CD163	Invitrogen	TNKUPJ
MerTK	Invitrogen	DS5MMER
Siglec1 = CD169	Invitrogen	SER-4
CD177	R&D Systems	1171A
Streptavidin	BD	564923 (Article number)
goat anti-rabbit	Thermo Fisher	A11035 (Article number)

### Biotinylation assay to determine RBC half-life

Five weeks post-transplantation, mouse RBCs from WT and *JAK2* mutant mice were labeled by intravenous injection of 1 mg EZ-Link™ Sulfo-NHS-Biotin (Thermo Fischer). Six days after biotin injection, mice were treated intraperitoneally 3 times a week for 2 weeks with an anti-mouse CD47 mAb (clone MIAP410; BioXCell) or a mouse IgG1 isotype control (clone MOPC-21; BioXCell) at a dose of 200 μg/mouse. For RBC cell half-life measurement, PB was obtained by sublingual vein bleeding at 48 h post-injection and then twice weekly for up to 5 weeks. For analysis, PB was stained with the following antibodies: anti-mouse Ter-119 (clone TER-119, eBioscience), Streptavidin (Biolgend), and SYTOX™ Blue Dead Cell Stain (Invitrogen). Stained cells were analyzed using the BD LSRFortessaTM cell analyzer.

### In vitro phagocytosis assay

Spleen cells were harvested from WT and *JAK2* mutant mice and macrophages were generated by culturing the cells in IMDM (Invitrogen) with 10% FBS and murine M-CSF (PeproTech) for 6 days. Macrophages were detached and plated in a 24-well plate with 2.5 × 10^5^ cells per well with 1 ml medium 1 day prior to the assay. The following day, RBCs from PV mice were incubated with CellTrace™ Yellow (Invitrogen) according to the manufacturers protocol. The RBCs were added to the macrophages at an effector to target ratio of 1:10 and the anti-mouse CD47 mAb (clone MIAP410; BioXCell) or a mouse IgG1 isotype control (clone MOPC-21; BioXCell) were added at a concentration of 5 μg/ml. The macrophages and RBCs were co-incubated for 2 h, then washed, stained, and analyzed. Phagocytosis of RBCs was defined by flow cytometry as the percentage of CellTrace™ Yellow-labeled cells out of F4/80+Aqua- cells.

### Statistical analysis

All statistical analysis was performed on GraphPad Prism 9 (Version 9.4.1). If the data were normally distributed, statistical analysis was performed as follows. Comparisons between two groups: unpaired *t*-test. Comparisons between more than two groups with unequal variance: Brown-Forsythe and Welch one-way ANOVA followed by Dunnett T3 post-hoc test. Comparisons between groups split on two independent variables on raw or transformed data: two-way ANOVA for multiple group comparison analysis of variance with post-hoc Tukey correction. Alternatively, Mann−Whitney and Kruskal–Wallis test with Dunn’s multiple comparisons were used if the data were not normally distributed. *P* values were as follows: ns not significant, *p < 0.05, **p < 0.01, ***p < 0.001, ****p < 0.0001. Error bars represent mean ± standard deviation. The RBC half-life was calculated using a nonlinear fit (plateau followed by one-phase decay). We used linear correlation and regression to determine the relationship between the two variables.

## Results

### Anti-CD47 treatment in a PV mouse model corrects polycythemia

**Fig. 1:**
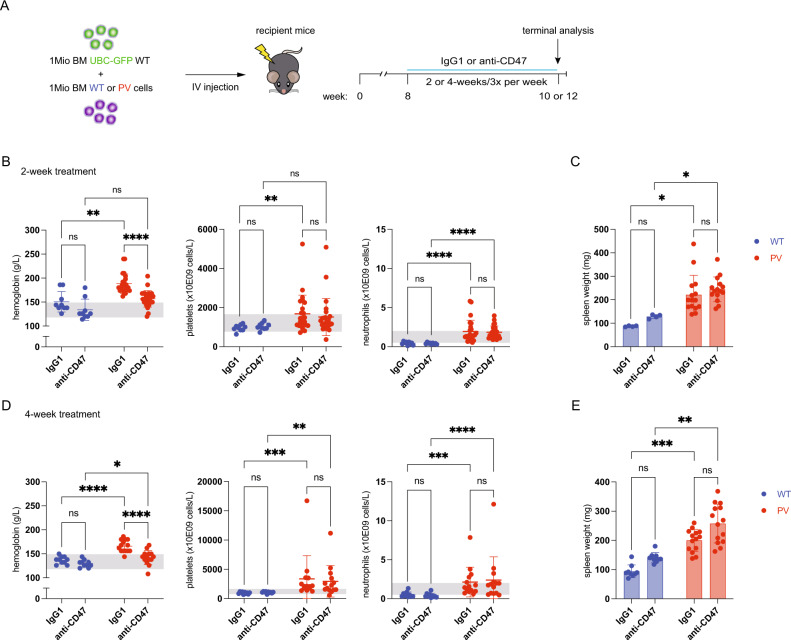
Anti-CD47 treatment in a PV mouse model corrects polycythemia. **A** Experimental workflow for the treatment of wild-type (WT) and *JAK2* mutant (PV) mice with an anti-IgG1 control (IgG1) or an anti-CD47 antibody. One million ubiquitin-GFP (*UBC-GFP*)-positive WT bone marrow (BM) cells were mixed with one million BM cells extracted from PV mice and transplanted into lethally irradiated WT recipients. Antibody treatment was performed 3 times a week for 2 or 4 weeks. Mice were sacrificed and analyzed after that. **B** Hemoglobin, platelet, and neutrophil counts (WT IgG1, *n* = 9; WT anti-CD47, *n* = 9; PV IgG1, *n* = 24; PV anti-CD47, *n* = 25) and (**C**) spleen weight (WT IgG1, *n* = 4; WT anti-CD47, *n* = 4; PV IgG1, *n* = 15; PV anti-CD47, *n* = 15) determined after 2 weeks of treatment. Gray shaded area indicates the normal range. **D** Blood counts and (**E**) spleen weight after 4 weeks of treatment. WT IgG1, *n* = 10; WT anti-CD47, *n* = 10; PV IgG1, *n* = 14; PV anti-CD47, *n* = 14. WT blue symbols, PV red symbols. Results are represented as mean ± standard deviation. ns not significant, *p < 0.05, **p < 0.01, ***p < 0.001, ****p < 0.0001 (Kruskal–Wallis test with Dunn’s multiple comparisons for **B** (hemoglobin and platelet counts), **C**, **D** (platelet counts), and **E**; two-way ANOVA with Tukey’s multiple comparisons test for **B** (neutrophil counts), and **D** (hemoglobin and neutrophil counts).

The expression of the pro-phagocytic (“eat me”) signal, CALR, was previously shown to be increased on human PV RBCs, while the anti-phagocytic (“don’t eat me”) CD47 ligand was reduced [24, 25]. We, therefore, first assessed the expression of CD47 and CALR on RBCs from a PV mouse model. Lethally irradiated recipient mice were transplanted with a 1:1 mixture of BM cells from GFP-positive WT mice and either WT or tamoxifen-inducible *Cre*-recombinase transgenic mice expressing the human *JAK2-*V617F transgene [26]. In line with the previous studies, we found reduced expression of CD47 and increased expression of CALR on PV RBCs (Supplementary Fig. 1A). To assess whether the disruption of the CD47-SIRPα interaction could lead to increased phagocytosis of PV RBCs compared to WT RBCs, we blocked the CD47-SIRPα interaction in the PV mouse model described above (Fig. 1A). Once the PV phenotype was established 8 weeks post-transplantation, mice were treated with an anti-CD47 antibody (clone MIAP410) or the IgG1 isotype control antibody (clone MOPC-21) for either 2 or 4 weeks. Consistent with previous studies [26], the expression of the *JAK2-*V617F transgene led to an increase of hemoglobin and RBCs in the peripheral blood (PB) (Fig. 1B, D and Supplementary Fig. 1B). At the same time, neutrophils and platelets were increased in PV mice (Fig. 1B, D). Anti-CD47 treatment significantly reduced hemoglobin and RBCs to almost normal levels in PV mice at 2 and 4 weeks of treatment, while platelet and neutrophil counts remained unaffected. PV mice developed pronounced splenomegaly, which was not affected by CD47-SIRPα blockade at 2 and 4 weeks in PV mice (Fig. 1C, E). In addition, the chimerism, defined by the proportion of GFP-negative cells, reached nearly 100% in PV mice compared to WT and was unaffected by anti-CD47 treatment (Supplementary Fig. 1C). These results indicate that anti-CD47 treatment primarily targets PV RBCs leading to a reduction in hemoglobin and RBCs in the PB.

### Abrogating SIRPα signaling is sufficient to correct the PV phenotype

**Fig. 2:**
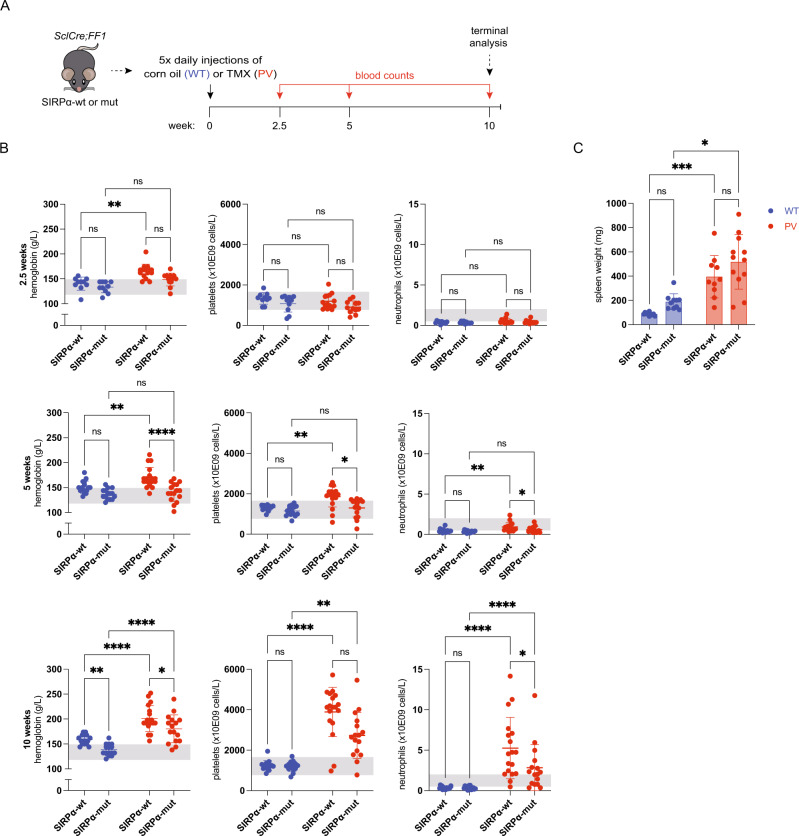
PV mice with absent SIRPα-signaling. **A** Experimental workflow assessing the impact of SIRPα-signaling in *SclCre;FF1 mice* on the SIRPα-WT (SIRPα-wt) or mutant (SIRPα-mut) background injected with corn oil (wild-type (WT)) or tamoxifen (TMX) (PV). WT SIRPα-wt, *n* = 10; WT SIRPα-mut, *n* = 10; PV SIRPα-wt, *n* = 10; PV SIRPα-mut, *n* = 12. **B** Peripheral blood counts 2.5-, 5- and 10-weeks post tamoxifen induction in mice with genotypes as indicated. Gray shaded area indicates the normal range. **C** Spleen weight. WT, blue symbols; PV, red symbols. Results are represented as mean ± standard deviation. ns not significant, *p < 0.05, **p < 0.01, ***p < 0.001, ****p < 0.0001 (Kruskal–Wallis test with Dunn’s multiple comparisons for **B** (2.5-week hemoglobin and neutrophil counts, 5- and 10-week platelet counts) and **C**); two-way ANOVA with Tukey’s multiple comparisons test for **B** (2.5-week platelet counts, 5- and 10-week hemoglobin and neutrophil counts)).

Antibodies unspecifically activate macrophages through antibody-dependent cellular cytotoxicity (ADCC) [27]. To rule out ADCC and show that the reduction of hemoglobin/RBCs described in the PV mouse model above was indeed the consequence of blocking the CD47-SIRPα interaction, we crossbred *JAK2* mutant mice with *Sirpα* mutant mice (hereafter called “*JAK2*-*Sirpα-*double mutant mice”) (Fig. 2A) [26, 28]. As observed with the antibody model, abrogating SIRPα signaling ameliorated the PV phenotype 2.5, 5, and 10 weeks after tamoxifen induction (Fig. 2B and Supplementary Fig. 2). In contrast to the antibody model (Fig. 1B), we also noted a reduction of platelets and neutrophils, particularly at later time points. Since CD47 is ubiquitously expressed and platelets expressing lower levels of CD47 are more prone to clearance by macrophages [29], the decrease of platelets and neutrophils in *JAK2*-*Sirpα-*double mutant mice is, as for RBCs, likely due to increased clearance by macrophages caused by the loss of the inhibitory SIRPα signal. As in the antibody model, *JAK2* mutant mice developed pronounced splenomegaly, which was not accentuated on the mutated *Sirpα* background (Fig. 2C). This shows the absent inhibitory SIRPα signal in *JAK2*-*Sirpα-*double mutant mice mediates the correction in hemoglobin levels.

### Anti-CD47 treatment marginally impacts PV RBC production

**Fig. 3:**
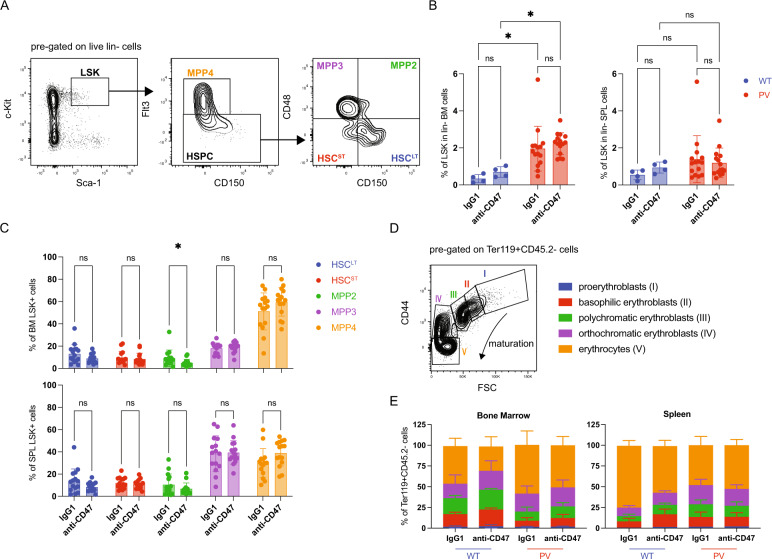
Impact of the anti-CD47 treatment on the production and maturation of RBCs in PV. **A** Gating strategy for the Lin-Sca-1 + c-Kit + (LSK) compartment and multipotent progenitors (MPPs), including long-term HSCs (HSC^LT^), short-term HSCs (HSC^ST^), MPP2, MPP3, and MPP4. PV IgG1, *n* = 15; PV anti-CD47, *n* = 15. **B** LSK fraction determined in the bone marrow (BM) and spleen (SPL). **C** Composition of the LSK compartment and MPPs in PV mice treated with IgG1 or anti-CD47 determined by flow cytometry at terminal analysis in the BM (top) and SPL (bottom). **D** Gating strategy for erythroid differentiation. Proerythroblasts (I; FSChi CD44hi), basophilic erythroblasts (II; FSCint CD44hi), polychromatic erythroblasts (III; FSCint CD44int), orthochromatic erythroblasts (IV; FSClo CD44int), and erythroblasts (V; FSClo CD44-). WT IgG1, *n* = 4; WT anti-CD47, *n* = 4; PV IgG1, *n* = 15; PV anti-CD47, *n* = 15. **E** Percentages of erythroid progenitors in the BM (left) and SPL (right) (% of FSC CD44 out of CD11b-CD45.2-Ter119+) determined by flow cytometry at terminal analysis. Results are represented as mean ± standard deviation. ns not significant, *p < 0.05 (Kruskal–Wallis test with Dunn’s multiple comparisons for **B** (BM LSK fraction); two-way ANOVA with Tukey’s multiple comparisons test for **B** (SPL LSK fraction); unpaired student’s *t*-test or Mann–Whitney test for **C**).

Given the role of macrophages in the production and the maturation of RBCs, we first assessed whether the reduction of RBCs in PV mice upon blocking the CD47-SIRPα interaction might be due to an impact of the treatment on the Lin-Sca-1 + c-Kit + (LSK) fraction, where the HSCs reside. As previously shown [26], the LSK fraction was increased in the BM of PV mice (Fig. 3A, B). However, anti-CD47 treatment did not affect the percentage of LSK cells in the BM and the spleen of PV mice. Within the LSK fraction, based on the expression of Flt3, CD48, and CD150, we further investigated HSCs and multipotent progenitors (MPPs), including long-term HSCs (HSC^LT^), short-term HSCs (HSC^ST^), MPP2, MPP3, and MPP4 [30]. Although the anti-CD47 treatment slightly reduced the MPP2 population in the BM of PV mice, the other MPPs remained unaffected (Fig. 3C). Next, we assessed RBC maturation in the BM and the spleen since, during steady and stress erythropoiesis, both BM and splenic macrophages support the maturation of erythroid progenitors in erythroblastic islands. Anti-CD47 treatment did not influence the distribution of erythroid precursors (Fig. 3D, E). In summary, the absence of CD47-SIRPα interactions marginally impacted the production of RBCs in the BM of PV mice.

### Splenic Mdcs expand upon anti-CD47 treatment in PV mice

We next investigated whether the normalization of hemoglobin and RBC levels may also be the result of increased degradation of PV RBCs. To address this aim, we performed a biotinylation assay, where mice were injected with biotin and, after 6 days, treated with the anti-CD47 antibody or the IgG1 isotype control antibody for 2 weeks (Supplementary Fig. 3A). Anti-CD47 treatment reduced the half-life of RBCs in both WT and PV mice compared to the IgG1 control (Supplementary Fig. 3B). This shows that anti-CD47 treatment leads to a reduction in RBC half-life that parallels the decrease in hemoglobin level observed in mice treated with anti-CD47 (Fig. 1B).

**Fig. 4:**
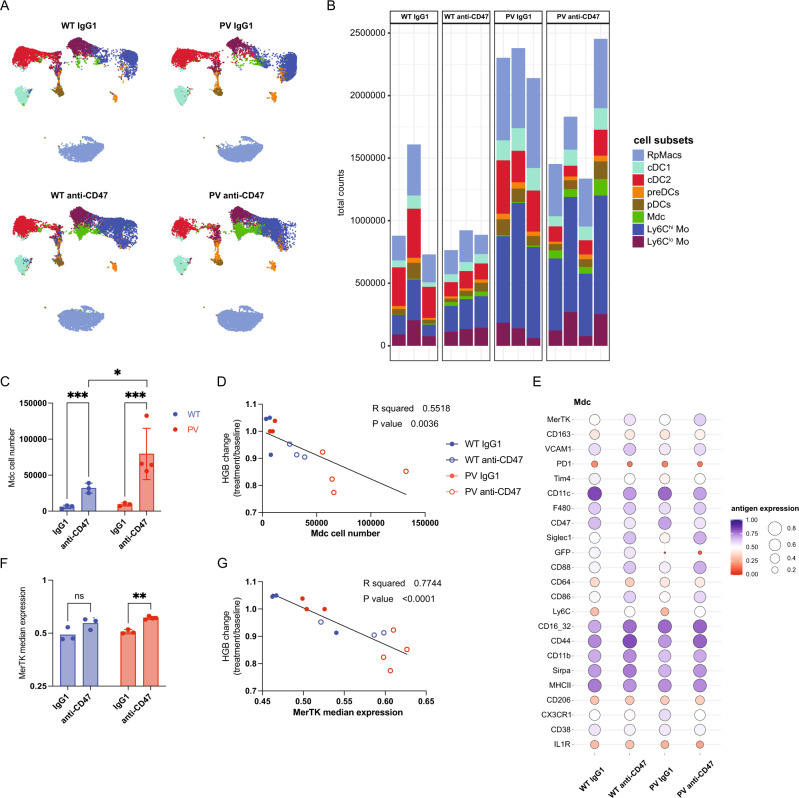
Splenic myeloid cells contribute to the therapeutic effect of anti-CD47 treatment in PV mice. **A** UMAP with FlowSOM overlay of total live CD45.2 + Lin-Neutrophil-Eosinophil- cells. 18,000 cells of combined samples are shown per group. Wild-type (WT) and *JAK2* mutant (PV) mice were treated with an anti-IgG1 control (IgG1) or an anti-CD47 antibody. WT IgG1, *n* = 3; WT anti-CD47, *n* = 3; PV IgG1, *n* = 3; PV anti-CD47, *n* = 4. **B** Bar plot depicting the total count of indicated FlowSOM-generated subpopulations per sample. Each bar represents one mouse. **C** The total count of FlowSOM-generated monocyte-derived effector cell (Mdc) population, shown per group. **D** Correlation of the hemoglobin change (treatment/baseline) to the Mdc cell number. **E** Antigen expression of indicated markers in FlowSOM-generated Mdc population, shown per group. The color and the circle size represent the mean of the median antigen expression of all samples per group. **F** Median expression and 25th and 75th percentiles of MerTK in FlowSOM-generated Mdc population. **G** Correlation of the hemoglobin change (treatment/baseline) to the medium expression of MerTK. WT, blue symbols; PV, red symbols. Results are represented as mean ± standard deviation. ns not significant, *p < 0.05, **p < 0.01, ***p < 0.001 (two-way ANOVA with Tukey’s multiple comparisons test for **C**; linear correlation and regression for **D** and **G**; Brown–Forsythe and Welch one-way ANOVA followed by Dunnett T3 post-hoc test for **F**).

Myeloid cells, predominantly macrophages, contribute to the degradation process of RBCs in the spleen, the primary site of RBC clearance. Therefore, we characterized the composition of myeloid cells in the spleen of WT and PV mice upon IgG1 or anti-CD47 treatment using high-dimensional single-cell flow cytometry combined with algorithm-guided analysis. The chimerism reached nearly 100% in PV mice compared to WT and was unaffected by anti-CD47 treatment (Supplementary Fig. 4B); therefore, extraction of GFP-negative cells was unnecessary for downstream analysis. We focused on monocytes, Mdcs, dendritic cell subsets, and macrophages (Fig. 4A, B and Supplementary Fig. 4A). Ly6C^hi^ inflammatory monocytes and red pulp macrophages (RpM) were significantly more abundant in PV than in WT mice (Supplementary Fig. 4C). Anti-CD47 treatment significantly increased Mdcs in PV mice compared to WT mice (Fig. 4C). At the same time, anti-CD47 treatment reduced RpMs in both WT and PV mice (Supplementary Fig. 4C). When we correlated the hemoglobin change (treatment/baseline) to the Mdc cell number, the reduction in hemoglobin from baseline significantly correlated with a higher number of Mdcs in PV mice treated with anti-CD47 (Fig. 4D). We next characterized the surface expression profile of Mdcs, Ly6C^hi^ monocytes, and RPMs. Upon anti-CD47 treatment, we observed an increased expression of the phagocytic marker MerTK on Mdcs from PV mice. In contrast, no phenotypic difference was observed on Ly6C^hi^ monocytes and RpMs between the groups (Fig. 4E, F and Supplementary Fig. 4E). Congruently, the expression of MerTK significantly correlated with the hemoglobin change (treatment/baseline) (Fig. 4G). Irrespective of anti-CD47 treatment, Mdcs in PV mice showed higher CD64 expression, a marker upregulated on macrophages only under pro-inflammatory conditions (Supplementary Fig. 4D) [31]. Given that under inflammatory conditions, Ly6C^hi^ monocytes are recruited to the site of inflammation and then have the potential to differentiate into Mdcs, we wanted to determine the impact of anti-CD47 treatment on their circulation. We found that PV mice had a significantly higher number of circulating monocytes in the PB, which was not influenced by anti-CD47 treatment (Supplementary Fig. 5). This data indicates that anti-CD47 treatment results in an increase of MerTK+ splenic Mdcs in PV mice. Considering the minor effect of anti-CD47 treatment on PV erythropoiesis, this infers augmented splenic degradation of PV RBCs as the main mechanism for the reduction of hemoglobin/RBC levels in PV mice.

### *JAK2* mutant macrophages are more “pro-phagocytic”

**Fig. 5:**
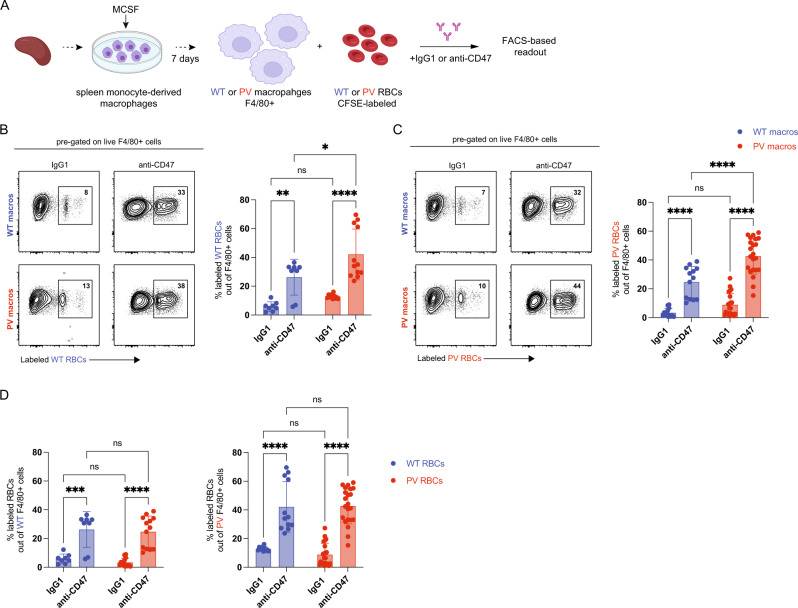
PV macrophages are more pro-phagocytic. **A** Experimental workflow for the in vitro phagocytosis assay of wild-type (WT) and *JAK2* mutant (PV) macrophages with WT or PV RBCs treated with an anti-IgG1 control (IgG1) or an anti-CD47 antibody. Created with BioRender.com. Percentage of labeled WT RBCs (**B**) and PV RBCs (**C**) out of F4/80+ spleen macrophages from WT and PV mice either treated with IgG1 or anti-CD47. Representative flow cytometry plots are shown on the left. **D** Percentage of labeled WT RBCs and *JAK2* mutant RBCs (PV RBCs) out of WT (left) and PV (right) F4/80+ spleen macrophages either treated with IgG1 or anti-CD47. WT, blue symbols; PV, red symbols. Results are represented as mean ± standard deviation. ns not significant, *p < 0.05, **p < 0.01, ****p < 0.0001 (two-way ANOVA with Tukey’s multiple comparisons test for **B**–**D**).

Somatic mutations in *JAK2* and other epigenetic regulators, such as *TET2* and *ASXL1*, prevalent in myeloid cancers, such as PV, induce proinflammatory conditions in monocytes and macrophages [25, 32, 33]. However, the impact on phagocytosis is unknown. To further investigate the degradation process of RBCs in the absence of CD47-SIRPα interactions, we investigated the phagocytic potential of clonal *JAK2* mutated splenic macrophages by performing an in vitro phagocytosis assay using spleen monocyte-derived macrophages from WT and PV mice. WT and *JAK2* mutant macrophages were co-incubated with either CellTrace™ Yellow-labeled WT or PV RBCs and treated with IgG1 or anti-CD47 (Fig. 5A). Phagocytosis of WT and PV RBCs was significantly increased in the presence of *JAK2* mutant macrophages compared to WT macrophages (Fig. 5B, C). Furthermore, no difference in susceptibility to phagocytosis was observed between WT and PV RBCs when exposed to either WT or *JAK2* mutant macrophages (Fig. 5D). This data indicates that somatic mutations, in this case, the *JAK2* mutation, can enhance the phagocytic potential of macrophages and likely contributes to the observed reduction of hemoglobin/RBCs in PV mice without CD47-SIRPα interactions (Fig. 1B).

## Discussion

The interest in macrophage immune checkpoints has significantly risen, mainly due to the therapeutic potential of the CD47-SIRPα blockade in hematopoietic and solid tumors [34, 35]. However, little is known about the relevance of this interaction in myeloproliferative neoplasms. Here, we show that blocking the CD47-SIRPα interaction results in the correction of polycythemia in a PV mouse model, which correlates with the expansion of splenic MerTK+ Mdcs and increased phagocytic activity of *JAK2* mutant splenic macrophages against RBCs. This data infers that PV RBCs undergo increased splenic degradation upon blockade of the CD47-SIRPα interaction.

The inflammatory state present in PV may support the efficient clearance of PV RBCs, which includes expansion and hyperactivation of phagocytes and increased secretion of pro-inflammatory cytokines, as reported for *JAK2* mutant murine macrophages [25]. We observed a significant increase of Ly6C^hi^ inflammatory monocytes in the spleen of PV mice and an increase of monocytes in the PB. Our findings further indicate that CD47 blockade leads to an expansion of phenotypically phagocytic MerTK+ splenic Mdcs in PV mice, which derive from monocytes. It has been shown that IFNγ and GM-CSF, which are increased in PV patients [36], can guide the transition of monocytes into Mdcs in experimental autoimmune encephalomyelitis, a model of tissue inflammation [8]. We, therefore, postulate that in our model, the addition of anti-CD47 further increases cytokine production, leading to the differentiation of Ly6C^hi^ monocytes into Mdcs [7, 37].

In addition, *JAK2* mutant macrophages also differ in their functionality. This behavioral change in clonal macrophages aligns with other reports that identified macrophages with *TET2* mutations, frequently found in myeloid neoplasms, to drive aberrant inflammation in vivo by releasing inflammatory cytokines [32, 38]. Other studies reported similar findings in monocytes carrying mutations in the other epigenetic regulators *DNMT3A* and *ASXL1* [33, 39]. These data highlight the importance of understanding the functional repercussions of somatic mutations in phagocytic immune cells and how this may affect neighboring immune cells.

Anemia is the most frequent adverse event reported in patients treated with anti-CD47 antibodies [35, 40, 41]. CD47 blockade can accelerate the clearance of aging RBCs due to the uncovering of pro-phagocytic signals, such as CALR [42]. Our study in a transgenic mouse model reproduces this on-target effect and yields further insights into the mechanism of anti-CD47-induced anemia. Interestingly, in PV, as shown by previous studies, RBCs reduce CD47 expression while increasing the expression of CALR, which would further drive their elimination upon CD47 blockade [24, 25]. Instead, our data shows that PV RBCs are not more susceptible to phagocytosis than WT RBCs, and the *JAK2* mutated phagocytic cells enhance their clearance. Furthermore, inflammation and clonal phagocytic cells can impact the efficiency of CD47 blockade since, in combination, they can contribute to the expansion of Mdcs. Presumably, Mdcs play a crucial role in this process and determine the severity of anemia in anti-CD47 treated patients. Anti-CD47 antibodies are currently being clinically investigated in patients with myeloid neoplasms, in particular myelodysplastic syndrome and acute myeloid leukemia [43]. It will be of interest to functionally characterize phagocytic cells derived from the mutant myeloid clone in these patients since, as observed in this study, somatic mutations in macrophages may impact their phagocytic function and potentially the outcome of macrophage checkpoint inhibition in a clinical setting.

We also addressed whether a decreased hematopoietic stem and progenitor cells pool in the absence of CD47-SIRPα interactions may contribute to reduced RBC levels in this model. Indeed, studies have shown that PV disease-initiating HSCs significantly up-regulate CD47 compared to normal HSCs, which could make PV HSCs more susceptible to macrophage phagocytosis in the absence of CD47-SIRPα interactions. However, our data show that CD47 blockade does not impact PV HSCs but leads to a modest reduction of the MPP2 population in the BM of PV mice. MPP2 is a distant subset of myeloid-biased MPPs [30]. Despite having primary megakaryocytic potential, it has a strong erythroid potential and transient ability to produce platelets. Hence, its reduction in the absence of CD47-SIRPα interactions may, together with increased degradation, contribute to the RBC reduction observed in PV mice.

In summary, our results show that anti-CD47 treatment expands splenic PV Mdcs with a phagocytic phenotype and that *JAK2* mutant macrophages are functionally “pro-phagocytic” and contribute to correcting the PV phenotype in the absence of CD47-SIRPα interactions. These findings highlight the importance of understanding the behavior of clonal macrophages in the pathogenesis of PV and potentially other malignancies.

## Supplementary information

Supplementary appendix

## Data Availability

All data and reagents used are available upon request.
