# Supplementary material for: Blocking the CD47-SIRPα interaction reverses the disease phenotype in a polycythemia vera mouse model
Source: Leukemia. 2023 Apr 24;37(6):1277–86. doi: 10.1038/s41375-023-01903-2 (PMC10244168; doi:10.1038/s41375-023-01903-2)
Supplement: Supplementary file 1 — Supplementary appendix [file 41375_2023_1903_MOESM1_ESM.pdf]

Supplementary Appendix

Table of contents

Section **Page**

Supplementary Figures

Section	Page
Supplementary Figure 1	2
Supplementary Figure 2	3
Supplementary Figure 3	4
Supplementary Figure 4	5
Supplementary Figure 5	6

Supplementary Figures

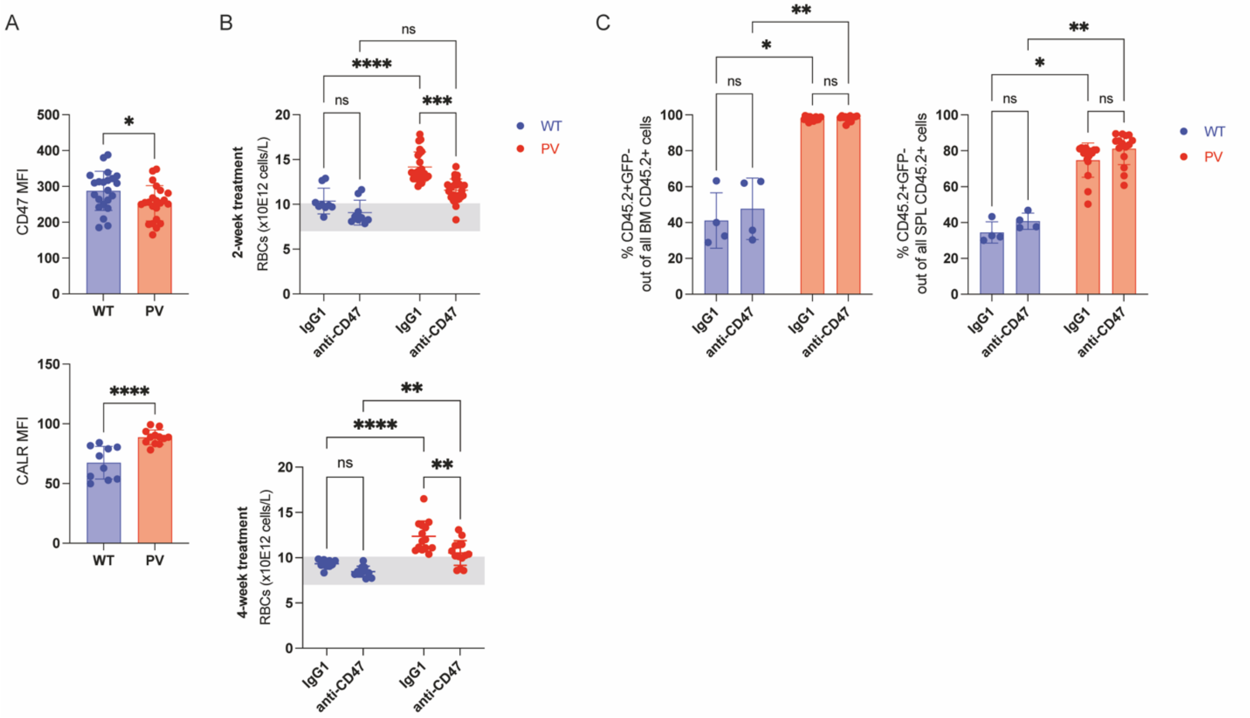

Supplementary Figure 1. (A) Mean fluorescence intensity (MFI) of CD47 (top) and CALR (bottom) on peripheral blood RBCs from wild-type (WT) and *JAK2* mutant (PV) mice. (B) RBCs of WT or PV mice treated with IgG1 or anti-CD47 for two (top) and four (bottom) weeks. Grey shaded area indicates the normal range. (C) Percentages of chimerism, determined by the proportion of CD45.2+GFP-, in the bone marrow (BM) and spleen (SPL) of treated WT and PV mice. WT, blue symbols; PV, red symbols. Results are represented as mean ± standard deviation. ns not significant, * p<0.05, *** p<0.001, *** p<0.001, **** p<0.0001 (unpaired student's t-test for A, Kruskal–Wallis test with Dunn's multiple comparisons for B (top) and C, and two-way ANOVA with Tukey's multiple comparisons test for B (bottom)).

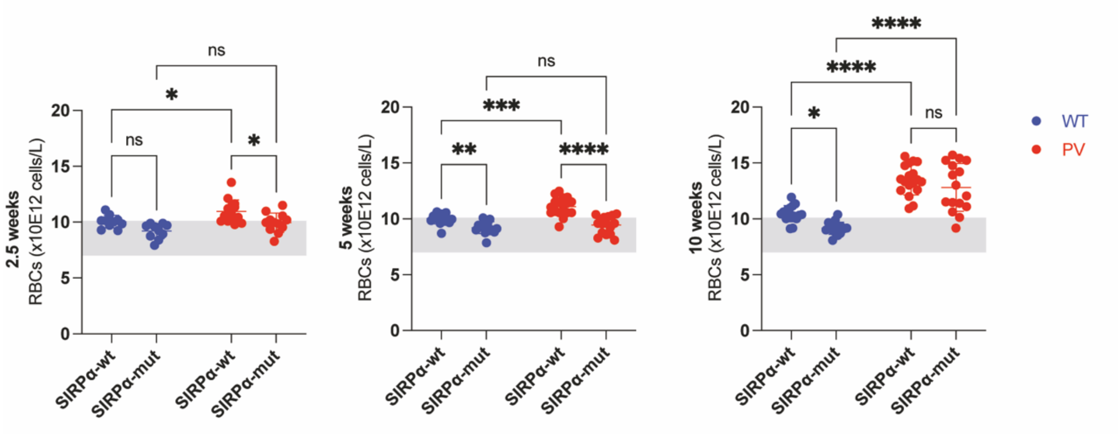

Supplementary Figure 2. RBC counts 2.5-, 5-, and 10-weeks post-tamoxifen induction of WT or PV mice on the SIRPα-WT (SIRPα-wt) or mutant (SIRPα-mut) background. Grey shaded area indicates the normal range. WT, blue symbols; PV, red symbols. Results are represented as mean ± standard deviation. ns not significant, * $p<0.05$, ** $p<0.01$, *** $p<0.001$, **** $p<0.0001$ (two-way ANOVA with Tukey's multiple comparisons test).

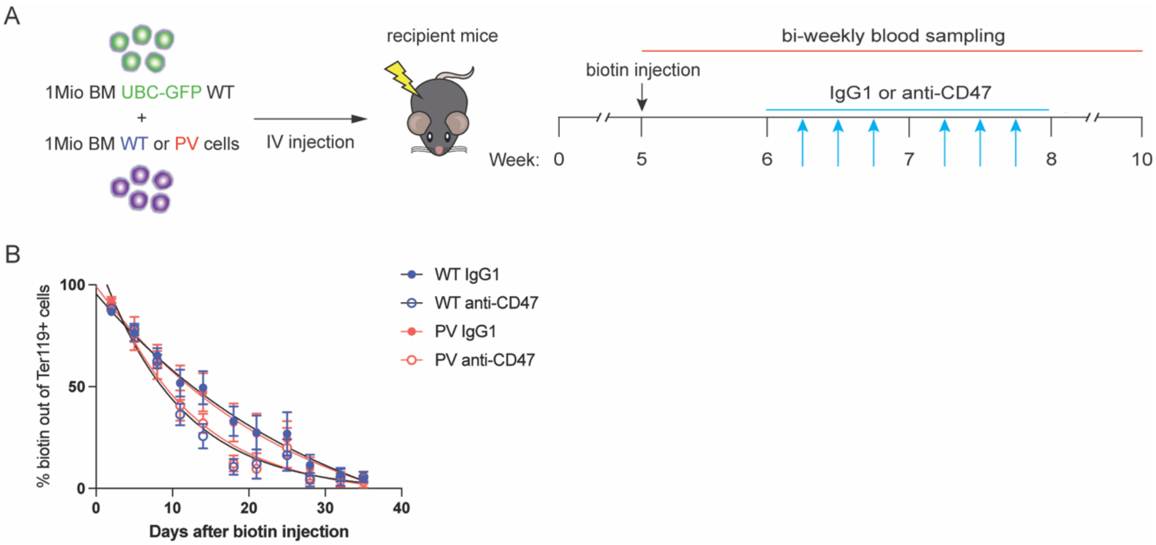

Supplementary Figure 3. (A) Experimental workflow for biotinylation assay of wild-type (WT) and *JAK2* mutant (PV) mice treated with an anti-IgG1 control (IgG1) or an anti-CD47 antibody. (B) Percentage of biotin out of live Ter119+ cells at 48h post-injection and then twice weekly for up to five weeks. WT, blue symbols; PV, red symbols. Results are represented as mean ± standard deviation (nonlinear fit (plateau followed by one phase decay)).

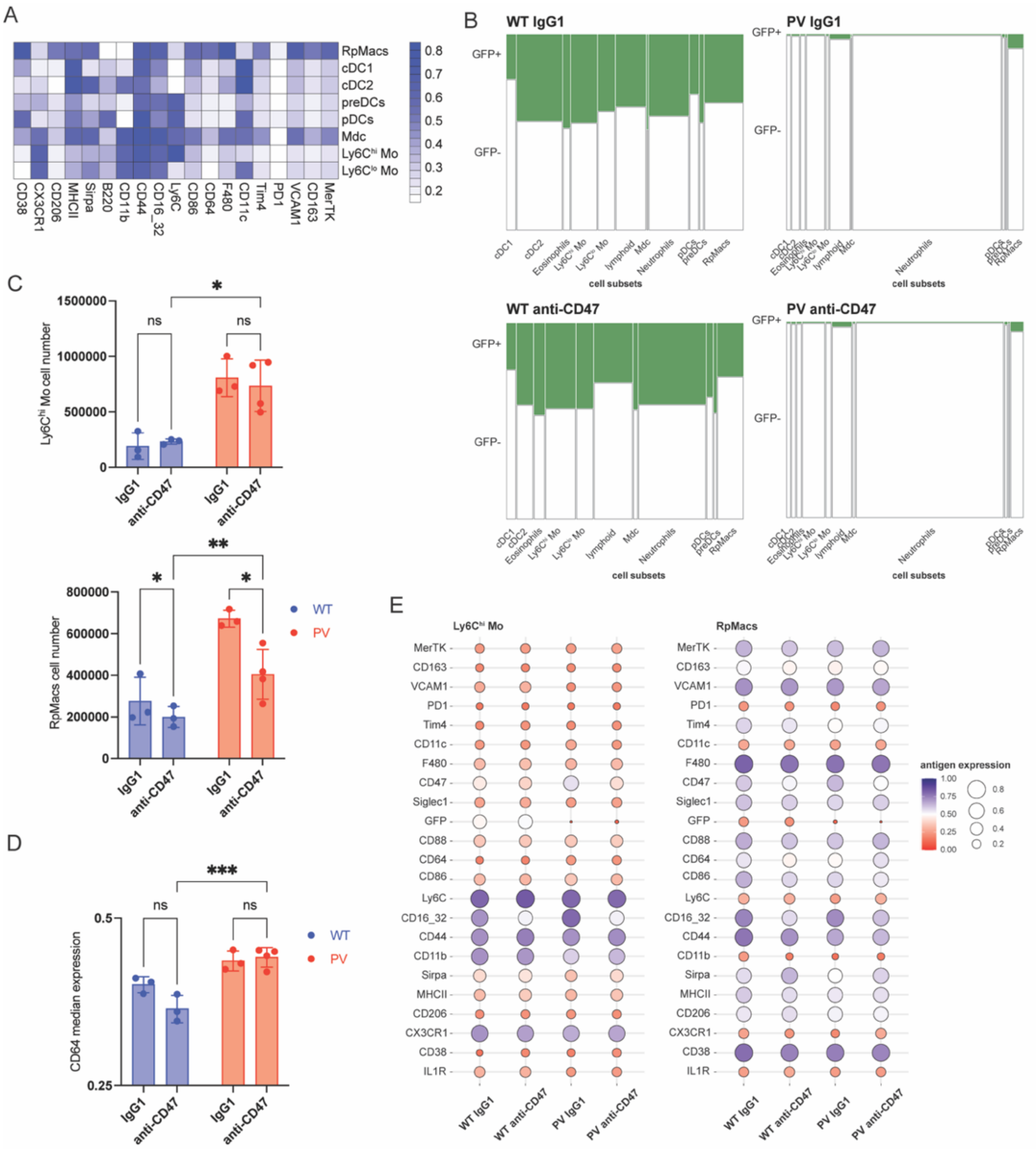

Supplementary Figure 4. (A) Median expression of various markers in FlowSOM-derived clusters shown in Figure 3A. (B) Mosaic plot depicting the chimerism of GFP+ (green) and GFP- cells (white) per FlowSOM-derived cluster in each group. Wild-type (WT) and *JAK2* mutant (PV) mice were treated with an anti-IgG1 control (IgG1) or an anti-CD47 antibody. The width of the bars represents the relative size of the population. (C) Total count of FlowSOM-generated Ly6C^{hi} monocyte (Ly6C^{hi} mo) and red pulp macrophage (RpMacs) population, shown per group. (D) Median expression and 25th and 75th percentiles of CD64 in FlowSOM-generated monocyte-derived effector cell (Mdc) population, shown per group. (E) Antigen expression of indicated markers in FlowSOM-generated Ly6C^{hi} monocyte and RpMacs population, shown per group. The color and the circle size represent the mean of the median antigen expression of all samples per group. ns not significant, * p<0.05, ** p<0.01, *** p<0.001 (two-way ANOVA with Tukey's multiple comparisons test for C and D).

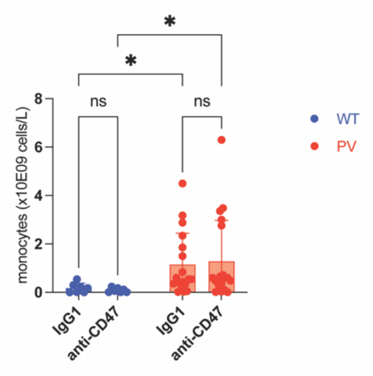

Supplementary Figure 5. Monocyte count in wild-type (WT) and *JAK2* mutant (PV) mice treated with an anti-IgG1 control (IgG1) or an anti-CD47 antibody after two weeks of treatment. WT, blue symbols; PV, red symbols. Results are represented as mean ± standard deviation. ns not significant, * $p<0.05$ (Kruskal–Wallis test with Dunn's multiple comparisons).
